# Genetic Variants in *SDC3*, *KCNA2*, *KCNK1*, *KCNK16,* and *Heat Shock Transcription Factor-1* Genes: An Exploratory Analysis Supporting the Piezo2 Channelopathy Hypothesis in Amyotrophic Lateral Sclerosis Onset

**DOI:** 10.3390/ijms262010218

**Published:** 2025-10-21

**Authors:** Balázs Sonkodi, Zsófia Flóra Nagy, Anikó Keller-Pintér, Péter Klivényi, Mária Judit Molnár, Márta Széll

**Affiliations:** 1 Department of Health Sciences and Sport Medicine, Hungarian University of Sports Science, 1124 Budapest, Hungary; 2 Department of Sports Medicine, Semmelweis University, 1122 Budapest, Hungary; 3 Department of Medical Genetics, Albert Szent-Györgyi Medical School, University of Szeged, 6720 Szeged, Hungary; 4 Institute of Genomic Medicine and Rare Disorders, Semmelweis University, 1085 Budapest, Hungary; 5 HUN-REN Functional Clinical Genetics Research Group, 6720 Szeged, Hungary; 6 Department of Biochemistry, Albert Szent-Györgyi Medical School, University of Szeged, 6720 Szeged, Hungary; 7 Department of Neurology, Albert Szent-Györgyi Medical School, University of Szeged, 6725 Szeged, Hungary; 8 HUN-REN-SZTE Neuroscience Research Group, Danube Neuroscience Research Laboratory, Hungarian Research Network, University of Szeged, 6725 Szeged, Hungary

**Keywords:** amyotrophic lateral sclerosis, whole exome sequencing, Piezo2, channelopathy, genetic variants

## Abstract

Amyotrophic lateral sclerosis (ALS) is a multisystem progressive neurodegenerative disease. A recent theory of ALS onsetting pathogenesis proposed that the initiating primary damage is an acquired irreversible intrafusal proprioceptive terminal PIEZO2 channelopathy with underlying genetic and environmental risk factors. This Piezo2 channelopathy may also disrupt the ultrafast proton-based oscillatory signaling to motor neurons through vesicular transporter 1 (VGLUT1) and to the hippocampus through VGLUT2. As a result, it may gradually degenerate motor neurons in which process K_v_1.2 ion channels are gradually depleted. It also gradually depletes heat shock transcription factor-1 (HSF-1) in the hippocampus, hence negatively affecting adult hippocampal neurogenesis. Syndecans, especially syndecan-3 (SDC3) in the nervous system, may act as critical players in the maintenance of the crosstalk between Piezo ion channels. Hence, our goal was to reanalyze the potential pathogenic gene variants from the cohort of our previous ALS study with a special focus on the aforementioned genes. Reanalysis of data formerly acquired by whole-exome sequencing of 21 non-related adult ALS patients was carried out with a focus on 28 genes. Accordingly, we identified charge-altering variants of SDC3 in 13 patients out of 21 that may contribute to the impairment of the Piezo crosstalk, and the progressive loss of the proposed proton-based signaling to motor neurons and to the hippocampus. A variant of uncertain significance was identified in the *KCNA2* gene that may facilitate the faster loss of K_v_1.2 ion function on motor neurons when Piezo2 channelopathy prevails. Not to mention that one variant was identified in the potassium current rectifying ion channels encoding *KCNK1* and *KCNK16* genes that may also propel the ALS disease process and provide the autoimmune-like pathogenic background. Moreover, Piezo2 channelopathy likely promotes diminishing HSF1 function in the hippocampus in the presence of the identified *HSF1* variant. The current findings may support the ALS onsetting acquired irreversible Piezo2 channelopathy-induced pathogenesis. However, the preliminary nature of these findings needs validation and further functional studies on cohorts with a larger sample size in the future.

## 1. Introduction

Amyotrophic lateral sclerosis (ALS) is a fatal neurodegenerative disease characterized by the loss of both upper and lower motor neurons. Only around a tenth of cases show a positive family history, while the other 90% remain categorized as sporadic disease. Despite this, sporadic ALS is appraised to have around 50% heritability, while for people without a known genetic risk, the estimate was lower (~36.9%) [1]. The current prevailing consensus is that ALS is rather an oligogenic disease; accordingly, only a few high-impact variants are present, but less impactful variants pave the way for disease development with the assistance of environmental factors [2,3]. ALS may also be considered a rare disease with an incidence of ~2.1–3.8 cases per 100,000 person-years in European populations [4]. Recent pooled estimates on ALS prevalence range from ~5–9 per 100,000, and a rising prevalence has been found to be associated with an aging population [5].

Currently, there is no effective treatment for most forms of ALS. However, an antisense oligonucleotide, tofersen, has been granted market authorization in the European Union, as recently as May 2024, in SOD1-associated ALS forms (https://ec.europa.eu/health/documents/community-register/html/h1783.htm—accessed on 15 November 2024). Drug development took a turn towards precision medicine in ALS, partially due to tofersen’s therapeutic success. Since the exact pathomechanism of ALS is yet to be fully understood, several approaches have been taken in order to better comprehend the pathological changes in ALS.

Our group has been focusing on the pathophysiology onsetting acquired Piezo2 channelopathy induced non-contact dying-back injury mechanism theory of ALS [6,7,8]. Excitotoxicity is also part of this theory, as has been previously named as a key component of neurodegeneration in ALS. Accordingly, the glial glutamate out-of-synapse transporter EAAT2 plays a pivotal role in the modulation of the excitatory overflow in a rodent model of ALS [9,10]. Therefore, reducing excitotoxicity has been proposed as a potential therapeutic intervention in neurodegenerative diseases [11]. The first drug to be indicated in ALS was riluzole, a noncompetitive blocker of N-methyl-D-aspartate (NMDA) receptors, which has been shown to significantly prolong the survival of ALS patients [12,13].

A genome-wide association study (GWAS) published in 2021 showed the abundance of ALS-associated risk loci in glutamatergic neurons. This data indicated that cell-autonomous neuron-specific processes initiate the degeneration in ALS [14]. This cell-autonomous excitotoxicity has also been shown in the *Drosophila melanogaster* model of C9orf72 hexanucleotide repeat expansion-associated ALS [15]. These findings may support the earlier suggested irreversible terminal microdamage theory of the glutamatergic Type Ia proprioceptive somatosensory neurons within the muscle spindle [8]. Later, it was even proposed that this terminal microdamage could be initiated autonomously by an acquired Piezo2 channelopathy in association with the impairment of the vesicular glutamate release machinery [8]. Correspondingly, the aforementioned GWAS also found perturbations in vesicle-mediated transport [14]. For years, there has been skepticism in regard to this autonomously acquired Piezo2 channelopathy theory, but lately, this microdamage of Piezo2 is emerging as a possibility [8,16,17,18,19]. Recently, it has even been suggested that overexcitation of Piezo2 on proprioceptive terminals under allostatic stress could dissociate accessory ligands, like MyoD-family inhibitor proteins or TMEM120A, leading to proton affinity switch-derived Piezo2 channelopathy [8]. In addition, oxaliplatin may also exert an analogous proton affinity switch, but directly on Piezo2 [19]. Further in support of the acquired nature of Piezo2 channelopathy that we found no inherited variances of *PIEZO2* among ALS patients from our previous reanalysis on the same cohort [7]. After all, we hypothesize that germline variants in genes associated with glutamatergic proprioceptive neurons and their function in proprioception and mechanostransduction could assist in the understanding of ALS pathomechanism.

Undeniably, the intrafusal Type Ia proprioceptive primary afferents contribute to alpha motor neuron degeneration [19,20]. Accordingly, it has been theorized that proprioceptive terminal Piezo2 microdamage-derived ‘switch’ of static phase firing encoding of Type Ia fibers to Type II fibers may result in a delay of the medium latency response (MLR) of the stretch reflex [8]. This ‘switch’ induced delay of MLR might have been verified in a delayed-onset muscle soreness (DOMS) study [21]. The proposed cause of the MLR delay is the impaired Type Ia afferent-derived monosynaptic static phase firing encoding input on motor neurons [21]. Moreover, Piezo2 channelopathy-induced impaired ultrafast proton-based signaling through vesicular glutamate transporter 1 (VGLUT1) may underpin this impaired input [18]. On the side of this impairment, a mouse model of ALS showed early dysfunctional abnormalities on neuromuscular junctions, leading to postsynaptic structural detachment from the neuromuscular junctions prior to motor symptoms in ALS [22]. It is also suggested that the VGLUT1 disconnection induced impaired ultrafast proton-based signaling to motor neurons progressively wears out neuromuscular junctions of these motor neurons and degenerates them as an irreversible ‘switch’ derived proprioceptive miswiring in ALS [8]. Indeed, a very recent preprint manuscript shows that *PIEZO2* deletion caused a slight reduction in the rapidly adapting mechanosensitive currents with very fast activation while not affecting the sensitivity or incidence of mechanosensitive currents [23]. Accordingly, the current authors suggest that this feature of *PIEZO2* makes it the principal mechanosensory channel responsible for proprioception, as the Nobel laureate Ardem Patapoutian and his team implicated [24]. Furthermore, this feature of *PIEZO2* may also involve the proposed ultrafast proton-based signaling initiation, as was earlier theorized [8,18] and may be presented in the aforementioned significant delay in the MLR of the stretch reflex [6,21].

It is noteworthy that an analogous Type Ia proprioceptive afferent input ‘switch’ has been shown in the early disease phase of spinal muscular atrophy (SMA), another motor neuron neurodegenerative disease, like ALS [25,26]. It is revealed in SMA that diminished proprioceptive synaptic drive leads to motor neuron functional impairment through the reduction of potassium channel K_v_2.1 at their surface [27]. Noteworthy is that the reduced expression of potassium channels has also been linked to ALS in animal studies [28]. Indeed, a reduced mRNA level of another potassium ion channel, namely *KCNA2*, is demonstrated in ALS motoneurons as well [29]. Moreover, it has been shown recently that in mice, the KCNJ10 inwardly rectifying potassium channel is downregulated in ALS but not in healthy ageing. The resultant extracellular potassium ion elevation also drives the excitotoxicity and aids in neurodegeneration [30]. Certain genotypes of potassium channel genes have been associated with longer survival in ALS patients, thus indicating a role of genetic variance in these genes in ALS as well [31]. Based on these data, our focus shifted to the germline variants in rectifying potassium channel genes in ALS patients and their possible involvement in the disease processes.

**Figure 1:**
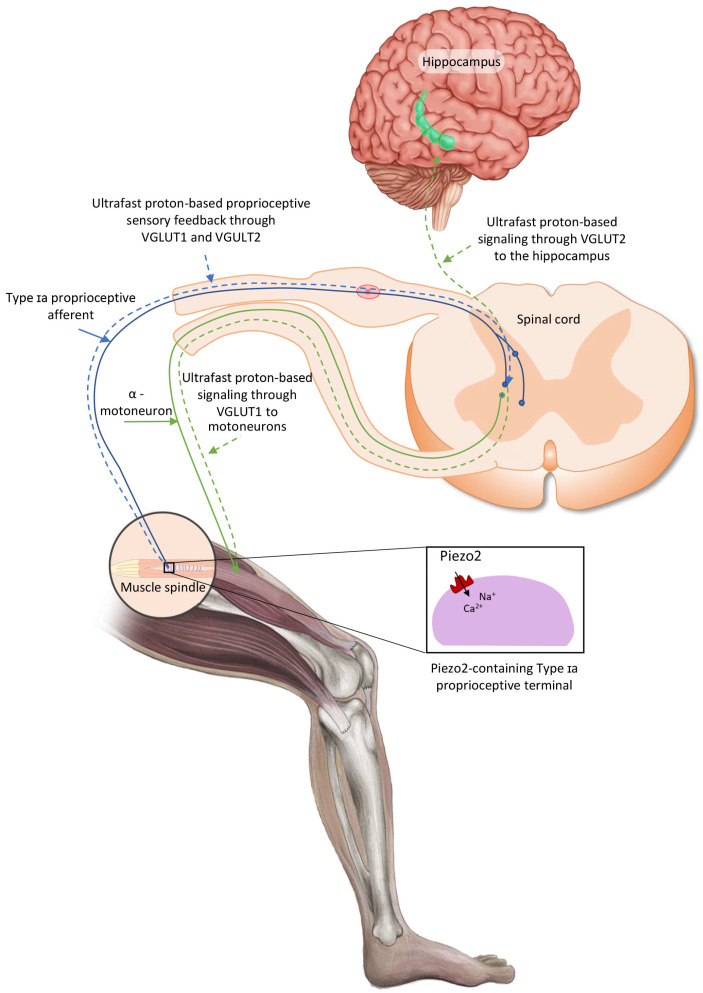
Proposed intrafusal proprioceptive terminal Piezo2 initiated ultrafast proton-based long-range synchronization signaling to hippocampal theta rhythm through vesicular glutamate transporter 2 (VGLUT2). The current figure is an English adaptation of the figure from Magyar Reumatológia [32]. Piezo2 channelopathy may impair this ultrafast proprioceptive signaling pathway, while the irreversible Piezo2 channelopathy may contribute to ALS onset.

In addition, the irreversible Piezo2 channelopathy theory of ALS posits that not only proton-based signaling to motor neurons is impaired through VGLUT1, but ultrafast proton-based long-range signaling to the hippocampus is also impaired through VGLUT2 (Figure 1) [8,18].

An interesting recent finding is that paired associative peripheral and transcranial electromagnetic stimulation reduced the symptoms of DOMS [33]. Correspondingly, it was suggested that this electromagnetic stimulation method revives the aforementioned transiently impaired ultrafast proton-based long-range neurotransmission to the hippocampus and to motor neurons in DOMS. Noteworthy is that Piezo2 was proposed to be the receptor for the detection of electromagnetic field-induced oscillating energy [8]. In support, a recent study indeed showed that *PIEZO2* has a critical role in the mediation of precise magnetic stimulation [34]. Moreover, repeated electromagnetic field stimulation (REMFS) has a positive effect on cellular senescence through heat shock transcription factor-1 (HSF1) by decelerating aging and death in cell culture [35]. Hence, we were interested in the encoding gene of *HSF1* in the ALS disease pathomechanism.

Moreover, not only Piezo2 initiated crosstalk between proprioceptive Type Ia terminal and hippocampus is impaired according to Piezo2 channelopathy theory, but disrupts the long suspected Piezo2-Piezo1 cross-talk in ALS as well [8,18]. In support, another recent finding demonstrated by localized mechanical stimulation is that Piezo2 mediates cell–cell communication through intercellular communication pathways [36]. Syndecans are likely a central first-line player of this Piezo2–Piezo1 crosstalk [8,37]. Syndecans constitute a four-member family of proteoglycans with negatively charged heparan and chondroitin sulfate chains. In addition to their role in various signal transduction pathways, the transmembrane localization establishes a physical link between the actin cytoskeleton and the extracellular matrix (ECM). The cytoplasmic domain of syndecans has a well-defined structure, and the ectodomains are intrinsically disordered, which is linked to a capacity to interact with multiple partners [38]. The expression of syndecans exhibits a characteristic pattern that is cell-, tissue-, and developmental stage-specific. Each cell presents at least one member of the syndecan family, and it is notable that they have redundant functions in order to compensate for each other [39]. Among the syndecan family, syndecan-3 presents the largest extracellular domain with three heparan sulfate and two chondroitin sulfate chains. The prominent presence of syndecan-3 in the central nervous system (CNS) has long been noted, while its role and functionality at the periphery are now being elucidated [40]. The key role of syndecan-3 in actin cytoskeleton-dependent processes has been well documented, e.g., in cell adhesion, migration, and neurite outgrowth of neurons [41]. In addition, syndecan-3 is a functional player in satiety control, spatial memory encoding [40,42], but they are also involved in inflammation and angiogenesis of certain diseases [40]. Syndecan-3 (SDC3) is also expressed in the regenerating nerve tissue and interacts with glial cell line-derived neurotrophic factor (GDNF) [43]. An important finding is that nerve injury of primary afferents upregulates syndecan-1 expression of these neurons in the dorsal root ganglion (DRG) [44]. Nevertheless, it was suggested to be a compensatory mechanism in response to autogenic syndecan-3 depletion or their functional loss, since syndecan-1 and syndecan-3 have redundant features [8,18].

Consequently, the primary goal of our study was to test our hypothesis in regard to the aforementioned acquired irreversible Piezo2 channelopathy theory of ALS by reanalyzing the potential pathogenic gene variants from our previous ALS study [45], with a special focus on the syndecan encoding genes, especially syndecan-3. Interestingly, syndecan-3 is implicated in the pathology of Alzheimer’s disease [46] and theorized in ALS [18], but no evidential relation has been reported yet. It is important to note again that our earlier reanalysis of the potential pathogenic gene variants from the same ALS cohort confirmed the absence of pathogenic variants of *PIEZO2* and *PIEZO1*, hence substantiating that the proposed irreversible Piezo2 channelopathy is acquired and not inherited [7], as theorized by the non-contact dying-back injury mechanism theory of ALS [6]. Furthermore, our reanalysis also had the purpose to examine the following genes: *CA1*, *CA2*, *CA3*, *CA4*, *CA9*, *VCAN*, *ACAN*, *ASIC2*, *ASIC3*, *SLC17A7*, *SLC17A6*, *KCNA2*, *KCNK* gene family, *TMEM120A*, MyoD-family inhibitor proteins encoding *MDFIC*, *MDFI,* and *MyoD1* genes, and *HSF1*.

## 2. Results

### 2.1. Analysis of the Syndecan Encoding SDC1, SDC2, SDC3 and SDC4 Genes

The reanalysis of WES data of 21 ALS patients revealed four *SDC3* variants altogether. The *SDC3* (c.G76C) p.G26R variant was identified in homozygous form in a male patient. The variant is located in the extracellular domain of the protein, and the establishment of a new, larger, and charged amino acid could confer negative consequences to the folding of the protein. The variant is currently classified as a likely benign sequence alteration.

Another variant affecting the charge of the amino acid was uncovered in 12/21 patients; the variants were in the heterozygous form in 10 patients and the homozygous form in 2 patients. Even though the *SDC3* (c.G907A) p.D303N variant is graded benign due to the high population frequency and was proven to have no pathogenic effect, it alters the charge of the 303rd amino acid position from negative to uncharged [47].

The additional two variants were the following: *SDC3* (c.G622A) p.V208I in 13 patients and *SDC3* (c.G286A) p.A96T in a single patient. Both of these variants are classified as benign.

Table 1 reports the most important data about the detected *SDC3* variants. It is noteworthy that most variants show a higher frequency in our small sample than the populational allele frequency.

### 2.2. Analysis of the Carbonic Anhydrase Encoding CA1, CA2, CA3, CA4 and CA9 Genes

The reanalysis of the carbonic anhydrase encoding *CA1*, *CA2*, *CA3*, *CA4,* and *CA9* genes detected no variants in the *CA1*, *CA2*, and *CA4* genes. A benign variant, the CA3 (c.G91A) p.V31I variant, was found in all 21 Hungarian patients in either heterozygous or homozygous form. Interestingly, the variant seems to be the common allele in the Hungarian population. A total of four *CA9* variants were uncovered, all of which are categorized as benign sequence alterations.

### 2.3. Analysis of the Versican and Aggrecan Encoding VCAN and ACAN Genes

The reanalysis of *VCAN* and *ACAN* genes resulted 21 different variants altogether. Out of the 21 various sequence alterations, all but one are considered benign with no predicted consequences. The uncovered variants of unknown significance (VUSs) in our study are only graded as a VUS, based on the low population frequency in populational databases despite sufficient coverage. The *ACAN* (c.T2902C) p.S968P variant has been submitted to ClinVar as a VUS.

Altogether, 47 rare missense variants have been reported in the *ACAN* gene in ALS patients in the project MinE database [48]. Out of the 47 rare missense variants, only 35 may be categorized as VUS. However, the VUS classification is mostly based on the population frequency of the variant and not on functional studies or disease association. No genomic constraint was noted in the case of the *ACAN* genes.

### 2.4. Analysis of ASIC2 and ASIC3 Encoding Genes

Three different variants in the *ASIC2* gene and one rare missense variant in the *ASIC3* gene were detected during the reanalysis. Out of the three *ASIC2* gene variants, two are rare variants, not found in comprehensive population genetic databases. Despite the rarity of these variants, all of them are either categorized as benign or a VUS leaning toward benign. Thus, none of them is expected to play a role in pathological processes.

### 2.5. Analysis of the VGLUT1 and VGLUT2 Encoding SLC17A7 and SLC17A6 Genes

The reanalysis revealed no variants in these two genes.

### 2.6. Analysis of the KCNA2 Gene and the KCNK Gene Family

A targeted re-analysis of the *KCNA2* gene in 21 ALS patients revealed a pathogenic-leaning VUS in the *KCNA2* gene (Table 2). The *KCNA2* (NM_004974.4) c.1351T>C; p.S451P variant changes an evolutionary conserved amino acid position close to the C-terminal of the protein. The variant is missing from the population genetic databases despite sufficient regional coverage. Most in silico predictors using a comprehensive algorithm support its pathogenicity. The variant has not yet been submitted to ClinVar.

No rare variants in the *KCNA2* gene are currently included in the project MinE database [49]. In total, 22 different variants were revealed during the screening of the KCNK gene family encoding the tandem pore domain potassium channels. Out of the 22 variants, 4 may be categorized as a benign-leaning VUS, and two pathogenic-leaning VUSs were detected. The KCNK1 (NM_002245.4):c.2T>A; p.M1K variant may result in a translational start loss due to the alteration of the starting codon. The variant has not been identified in the healthy population, and several prediction databases support its pathogenicity (SIFT, MutationTaster, DANN). The KCNK16 (NM_001135106.2):c.502C>T; p.Q168TER is a stop-gain variant not described in population databases before. The variant is located towards the C-terminal of the protein and may result in the loss of two exons.

During the screening of the *KCNK* gene family in the project MinE database [49] we identified 13 VUSs in the *KCNK1* gene, 13 VUSs in the *KCNK2* gene, 16 VUSs in the *KCNK3* gene, 24 VUSs in the *KCNK5* gene, 22 VUSs in the *KCNK6* gene, 19 VUSs in the *KCNK7* gene, 14 VUSs in the *KCNK12* gene, 29 VUSs in the *KCNK13* gene, 17 VUSs in the *KCNK15* gene, 18 VUSs in the *KCNK16* gene, and 16 VUSs in the *KCNK17* gene. A likely pathogenic variant and 21 further VUSs were identified in the *KCNK18* gene. No rare variants have been detected in the database in the *KCNK4*, *KCNK9,* and *KCNK10* genes.

The likely pathogenic c.234del (p.Asp78Glufs*13) variant in the *KCNK18* gene (NM_181840.1) is a single nucleotide deletion leading to a frameshift and to the formation of a premature stop codon (Table 3). According to the prediction, the truncated nucleotide strand will undergo nonsense-mediated decay and may act in a loss-of-function manner. The numerous VUSs must be interpreted with great caution, since not much data is available on their functional effect, and their classification is mainly based on the population frequency data and the result of prediction algorithms.

Interestingly, there is an increased burden of stop-gain variants in ALS patients in the project MinE database in the *KCNK6*, *KCNK15*, *KCNK16,* and *KCNK17* genes, but not in the other genes of the gene family. Upon closer analysis of the *KCNK16* gene, in which we identified a novel stop-gain variant (c.502C>T; p.Q168*), we detected another *KCNK16* stop-gain variant (c.278G>A; p.W93*) in exon 2 of the gene in the project MinD database, which shows an increased genomic burden among ALS patients. We hypothesize that stop-gain variants of the *KCNK6*, *KCNK15*, *KCNK16,* and *KCNK17* genes are more disruptive to the function of the ion channel than the stop-gain variants of other genes of the gene family. However, the pathomechanism of this hypothesized phenomenon remains elusive, and more research is needed to confirm or rule out the association (Supplementary Materials).

### 2.7. Analysis of TMEM120A Encoding Gene

The reanalysis of the NGS data focusing on the *TMEM120A* gene revealed two benignly classified synonymous variants (rs4732519 in all 21 patients and rs8509 in 2 patients).

### 2.8. Analysis of the MyoD-Family Inhibitor Proteins and MyoD Encoding MDFIC, MDFI, and MyoD1 Genes

The reanalysis revealed no variants in these three genes.

### 2.9. Analysis of HSF1 Encoding Gene

The reanalysis of the *HSF1* gene in our patient cohort resulted in the detection of a pathogenic-leaning VUS splicing variant (Table 3). The *HSF1* (NM_005526.4):c.861-2A>C variant affects the sequence just before the translational start of the 9th/13th exon of the gene. The variant is not found in any of the comprehensive population genetic databases and has not been included in ClinVar. SpliceAI predicts a very strong chance of a splice acceptor loss, which could lead to an intron retention in the translational process.

Thirty missense and splice site rare *HSF1* gene variants have been submitted to the project MinE database, which all hold the VUS classification [49]. As in the case of the *ACAN* gene variants, the pathogenicity was also determined based on sparse data, mainly the variant frequency. No genomic constraint was noted in the case of *HSF1* genes, as is the case in reference to *ACAN* genes.

## 3. Discussion

The current reanalysis is meant to test an ALS onset underlying mechanism hypothesis that is initiated by acquired irreversible proprioceptive Piezo2 channelopathies [6,7,8,18]. The transient form of this acquired Piezo2 channelopathy, implicated as the primary damage in DOMS [8,50], is suggested to microdamage the Piezo2 function of intrafusal primary afferent terminals in an autonomously acquired way under an acute stress response [8]. Moreover, this acquired Piezo2 channelopathy also proposed to impair the crosstalk between Piezo2 and Piezo1 channels in the given compartmental micromilieu and the crosstalk between Piezo2 and Piezo2 channels beyond the given compartments [8].

Syndecans, especially syndecan-3, are likely the central players of this Piezo crosstalk on proprioceptive neurons (Table 4) [8,18,37]. Hence, the current charge-altering variant findings of syndecan-3 could bear relevance in regard to the theoretical loss of Piezo2–Piezo1 cross-communication in ALS pathomechanism. An in-depth explanation of how syndecans and especially syndecan-3 may have a role in ALS pathophysiology can be found in a recent theoretical paper [18].

Earlier, it was also proposed that CA proteins have an essential role in the proton-signaled cross-communication between motoneurons and mechanotransducing proprioceptive glutamatergic neurons with Piezo2 content [18]. However, the suggested irreversible Piezo2 channelopathy may cause VGLUT1/Ia synaptic disconnection on motoneurons [7,50]. As a result, the impaired Piezo2-initiated resonance on the proprioceptive afferents may upregulate CA1 on the spinal level and consequently alter the distribution of the subpopulation of CA1 on the endoplasmic reticulum membranes of motor neurons in the affected compartmental micromilieu [18]. This feed-forward compensatory protein amplification may be due to the impaired Piezo2 signaling. Correspondingly, we analyzed but did not find any pathogenic variants of the *CA1*, *CA2*, *CA3*, *CA4*, and *CA9* genes; therefore, the functional compensatory role of CA1 might be substantiated.

Moreover, an analogous feedforward upregulation of ASIC2 in motoneurons of ALS is proposed to be the consequence of Piezo2 channelopathy [18]. Despite this compensatory mechanism, we were curious whether any potential pathogenic gene variants could be detected on the *ASIC2* encoding gene, but we found none. Noteworthy that the role of CA and ASIC2 in ALS pathophysiology could be familiarized from a current theoretical paper [18]. We also examined *ASIC3* encoding genes since ASIC3 is the secondary proprioceptive channel in compensation for the functional loss of Piezo2 [8], but we did not find any variants on this gene either.

Impairment of glutamate vesicular release [21] due to proton reversal is part of the Piezo2 channelopathy theory [8]. Indeed, protons are known to regulate the control of VGLUT proteins [51], hence the proton affinity switch and the resultant unidirectional proton leak may have a role in this impairment [8,19]. Noteworthy, VGLUTs also behave like proton-glutamate antiports [49]; therefore, the impairment of glutamate vesicular release could explain VGLUT synaptic disconnection on motoneurons, not to mention the impairment of the ultrafast long-range proton-based oscillatory synchronizing neurotransmission to the hippocampus [18]. Therefore, we were interested in detecting any pathogenic variants on *VGLUT1* and *VGLUT2*, but we did not find any.

Indicative research showed that chronic stress-derived muscle acidification leads to muscle mechanical hyperalgesia with the involvement of extracellular matrix proteoglycans and ASIC3 [52]. This research also showed that versican likely compensated for the initial syndecan loss on the chronic path downstream. Upregulation of versican is detected in the early symptomatic stage of ALS, but depletion is present in the late symptomatic stage [53]. Correspondingly, we analyzed the versican and aggrecan encoding *VCAN* and *ACAN* genes (Table 4), as we did on *ASIC2* and *ASIC3* genes (Table 4), but did not find any pathogenic variants. Hence, these negative results may support their functional feed-forward compensatory role in the ALS onset of irreversible Piezo2 channelopathy pathomechanism theory. In addition, it is important to note that the metabolism of collagen, as the most prominent ECM constituent, may also be affected downstream by this feed-forward compensatory mechanism in ALS, as has long been observed [54]. This might be especially critical with respect to the current theoretical pathophysiological framework since collagen bundles are known to be piezoelectric [55].

*The KCNA2* gene encodes the K_v_1.2 voltage-gated delayed-rectifier potassium channel that is involved in mechanotransduction (Table 4). The reduced expression of potassium channels has been implicated in animal models of ALS [28]. Accordingly, the reduced level of *KCNA2* mRNA was shown in ALS motoneurons [29]. Moreover, K_v_1.2 ion channels show early motoneuron axonal degenerative alterations in support of the dying-back pathomechanism theory of ALS, in a way that the central potassium channel function of these motoneurons is enhanced with increased amplitude and duration after hyperpolarization [56]. In support of the acquired irreversible Piezo2 channelopathy theory of ALS, the intrafusal group Ia afferents are highly represented by K_v_1.1 and K_v_1.2 ion channels, substantiating their role in proprioception, and they are suggested to contribute to their dynamic firing properties [57]. The current authors suggest that acquired irreversible Piezo2 channelopathy of Type Ia afferents not only irreversibly impairs static phase firing encoding of these neurons, leading to motoneural disruptions at motoneuron junctions [8,18], but leads to impaired or lost maintenance of delayed rectification of the static phase firing encoding. Consequently, K_v_1.2 ion channels may not be able to cope with slow inactivation. Important to note in support that a very recent preprint shows that *PIEZO2* deletion not only abrogates the rapidly adapting mechanosensitive currents with very fast activation, but also delays the very fast inactivation as well [23]. This insufficiency may lead to a decrease in K_v_1.2 ion channel function and hyperexcitability of motoneurons in a later, but still early, symptomatic stage of the ALS course. In support, K_v_1.2 ion channels are indeed reduced in the ventral roots of ALS patients, in contrast to the dorsal roots [58]. As a result, these reduced potassium currents in motoneurons of ALS patients further elevate hyperexcitability and may contribute to fasciculation generation [58]. Another study verified the disease stage-specific impairment of potassium channels, including K_v_1.2, and K^+^ conductance decline, but also showed preceding Na^+^ conductance increases [59]. After all, the found pathogenic-leaning VUS in the *KCNA2* gene may support the above observations and theory.

We also reported recently in our former reanalysis of the same ALS cohort that VUS in Na_v_1.1 encoding *SCN1A* genes are present [7]. Important to highlight again that *SCN1A*, *SCN8A*, and *SCN9A* genes encode the Na_v_1.1, Na_v_1.6, and Na_v_1.7 channels, and they are all present on proprioceptive afferents. The involvement of Piezo2 and Na_v_1.1 channels in the encoding of the static phase firing of proprioceptors has been shown [60,61]. In contrast, it is suggested that the combination of Piezo2, Na_v_1.6, Na_v_1.7, and/or other channels, such as glutamate receptors, ASIC, and ENaC ones, is responsible for the dynamic phase encoding of proprioception [60]. Thereby, the current authors propose that the irreversible impairment of Piezo2 function at proprioceptive terminals leads to increased Na^+^ conductance, not to mention that if VUS is present, then eventually it impairs delayed-rectifier potassium channels, like K_v_1.2, in ALS. Hence, K_v_1.2 ion channel decline not only impacts the dynamic phase firing encoding, but the static phase firing encoding as well. Hence, irreversible Piezo2 channelopathy may lead not only to loss of proton handling to ASIC2 [18] but also to enhanced intracellular cation influx of Ca^2+^ and Na^+^, which eventually leads to insufficient delayed rectification of K_v_1.2 ion channels on primary proprioceptors, hyperexcitability, and fasciculations.

An interesting earlier finding is that antibodies to L-type voltage-gated calcium channels appear in the serum of ALS patients, and titers of these antibodies correlate with the rate of disease progression [62]. Noteworthy is that we also reported from a previous reanalysis of the same ALS cohort that likely pathogenic variants of *CACNA1D* were found [7]. *CACNA1D* is the encoding gene of the Ca_v_1.3 L-type voltage-gated calcium channel, and is suggested to be important because its role in dysregulated pain pathways in ALS is likely due to irreversible Piezo2 channelopathy [7]. Hence, not only could antibodies make L-type voltage-gated calcium channels malfunctional, but likely pathogenic variants as well. Moreover, a higher level of voltage-gated potassium channel antibody titer was shown in a subset of ALS patients as well [63], further suggesting the autoimmune mechanism in the pathogenesis of ALS. Accordingly, acquired Piezo2 channelopathy has been theorized to be involved in autoimmune diseases with the involvement of dysfunctional K2P ion channels, even highlighting an immune-mediated dysfunction linking rheumatoid arthritis and ALS pathomechanism, based on shared disease-associated single-nucleotide polymorphisms from GWAS [37,64,65]. K2P is a mechanosensing and acid-sensing ion channel, and Piezo1/Piezo2 ion channels are known to foster its mechanogating [66]. Furthermore, K2P indeed has a role in autoimmune attacks within the CNS and neurodegeneration [67]. K2P is also called a background leak potassium ion channel due to its contribution to background potassium current rectification, and it has a key role in pressure overload injury-induced remodeling [68]. Imiquimod-induced inhibition of DRG neurons may be indicative of the co-functioning pathway of K_v_1.1, K_v_1.2, and K2P ion channels [69]. The current authors suggest that irreversible Piezo2 channelopathy induces progressive K_v_1.1, K_v_1.2, and K2P ion channels dysfunction in DRG neurons that may lead to loss of pressure overload-induced remodeling and to the initiation of the autoimmune mechanism of ALS pathogenesis. The found variants of uncertain significance on *KCNK1* and *KCNK16* encoding genes might also support the current theoretical framework.

The relevance of the differential expression pattern of MyoD is highlighted as the metabolic shift to oxidative metabolism underpinning the switch from fast to slow-twitch muscle in ALS disease progression, in order to sustain muscle function [70] and postural control [18]. Underpinning this mechanism, DOMS is suggested to involve a transient proprioceptive switch derived distal axonopathy, while ALS likely involves an analogous chronic, irreversible phenomenon [8]. MyoD-family inhibitor proteins are auxiliary subunits of Piezo1 and Piezo2 ion channels, and they present a functional control over Piezo inactivation [71]. Moreover, TMEM120A, or TACAN, also exhibits such a feature on Piezo2 [72]. However, prolonged stretch under allostasis may dissociate these auxiliary proteins from Piezo2, leading to a unidirectional inward proton path when this proton leak should not exist [8,19]. Furthermore, this proton leak on proprioceptive terminals may contribute to the impairment of oxidative phosphorylation (OXPHOS) and the astrocyte-neuron lactate shuttle-like machinery by switching glycolysis, or even more importantly, glutaminolysis in parallel, to energetically lower fermentation pathways [19]. Not to mention that this suggested proton leak not only causes Piezo2 channelopathy, but may also impair the vesicular glutamate release machinery that is needed for prolonged stretch signaling [19]. Accordingly, we analyzed the MyoD-family inhibitor proteins and MyoD encoding *MDFIC*, *MDFI,* and *MyoD1* genes, but did not find any pathogenic variants.

The *HSF1* gene encodes the heat shock transcription factor-1 that regulates several heat-shock proteins under stress. HSF1 is activated by various stressors, like heat shock, hypoxia, misfolding proteins, free radicals, and adenosine triphosphate shortage [73]. Earlier, the critical role of Hsp70/TLR4/Interleukin-6, TLR4/Myd88, and TNF-α pathways [74,75,76] was emphasized in the acute form of the suggested Piezo2 channelopathy-derived neuroinflammation, like is theorized in DOMS [50]. However, when Piezo2 channelopathy may become irreversible in ALS, the activated NF-κB pathway by TLR4/Myd88 signaling keeps on propelling the neuroinflammation in the CNS with non-resolving progressive impairment of the proprioceptive circuitry [77]. The heat shock response pathway is initiated by HSF1 activation and regulates Hsp70 expression [78]. Moreover, TNF-α transcription could be repressed by an HSF1 binding site [79]; hence, the variants of *HSF1* may lead to enhanced expression of TNF-α and may affect neuroinflammation in the CNS. Indeed, elevated level of TNF-α and its receptors is known in ALS [80]. In return, increased TNF-α may even decrease HSF1 activation [81]. HSF1 is in crosstalk with insulin signaling as well; hence, HSF1 activation could increase Hsp70, inhibit NF-κB activation-derived neuroinflammation, and increase insulin sensitivity [82]. In contrast, decreased HSF1 activation may reduce Hsp70 expression, increase NF-κB activation-derived neuroinflammation, and decrease insulin sensitivity. In support, HSF1 overexpression elongates life in a mouse model of ALS [83], as exogenous administration of Hsp70 as well [84]. Indeed, it has been demonstrated earlier that HSF1-induced upregulation of various Hsps was highly neuroprotective in ALS cell culture [85], and they also showed that failure to activate HSF1 resulted in a high threshold to induce stress response in motor neurons [86]. Furthermore, irreversible Piezo2 channelopathy-induced loss of Piezo2–Piezo1 crosstalk is proposed to increase insulin resistance and dysregulate glucose metabolism in ALS [18]. Noteworthy, HSF1 was demonstrated to induce high transcriptional activation of Hsp70 and Hsp40, leading to significant suppression of TDP-43 aggregation [87]. Hence, the HSF1-dependent clearance mechanism might also be impacted by the found variance. An interesting recent finding is that REMFS has a treatment effect on cellular senescence through HSF1 by decelerating aging and death in cell culture [35]. This impact is likely accomplished by proton tunneling, where REMFS acts like a driven quantum oscillator of interfacial water [35]. An analogous driven quantum oscillator function has been devoted to the Schottky barrier diode-like feature of Piezo2 via VGLUT2 in order to set up the suggested ultrafast proton-based long-range oscillatory synchronizational neurotransmission from the peripheral intrafusal proprioceptive terminal to the hippocampus [8,18]. Moreover, Piezo2 was proposed to detect electromagnetic field-induced oscillating energy [8]. Since Piezo2 channelopathy is suggested to be a principal transcription activator, it may not only impact adult hippocampal neurogenesis [19], but might activate non-coding heat shock RNA-1 (HSR1) in order to activate HSF1. However, these functions are lost in the suggested irreversible form of Piezo2 channelopathy, like in ALS. A recent significant finding is that *PIEZO2* is indeed the underlying mediator of precise magnetic stimulation [34], as was theorized earlier [8]. Even more importantly, the result of our current genetic reanalysis, namely the pathogenic-leaning VUS splicing variant of *HSF1*, might underline the *PIEZO2*-related aforementioned functional finding when it comes to precise magnetic stimulation.

## 4. Materials and Methods

An investigation of Hungarian ALS patients was carried out in 2018. Patients underwent thorough neurological examinations performed by senior neurologists. All patients exhibited both lower and upper motor neuron signs at the time of recruitment and fulfilled the Awaji-Shima and revised El-Escorial ALS diagnostic criteria at the time of examination [88,89]. No patient reported a positive family history for ALS or any neurodegenerative diseases. Within the framework of that project, samples of 21 non-related sporadic ALS patients underwent whole exome sequencing (WES). Table 5. contains additional demographic data concerning our 171 patients.

WES was carried out on an Illumina NextSeq 500 sequencing device (mean on-target coverage: 71× per base, 90% of targeted bases covered more than 10-fold). Paired-end reads were aligned to hg19. Variant calling and filtering process were carried out Genome Analysis Toolkit software (GATK) version 1.6-23.gf0210b3 (Broad Institute, Cambridge, MA, USA) and the ANNOVAR software version 2017. tool for annotation of the called variants. Variants with a low variant allele frequency (VAF < 0.3), poorly covered targets (read depth of <10) were excluded from the analysis. Variant classification was carried out in accordance with the 2015 guidelines of the American College of Medical Genetics [90].

The analysis workflow is described in more detail in our 2019 publication [45]. In possession of the previously acquired whole-exome sequencing data, we opted for the reanalysis of the dataset, focusing on genes not assessed before.

A targeted reanalysis of the formerly acquired data was carried out in order to explore the potential involvement of the *SDC1*, *SDC2*, *SDC3*, *SDC4*, *CA1*, *CA2*, *CA3*, *CA4*, *CA9*, *VCAN*, *ACAN*, *ASIC2*, *ASIC3*, *SLC17A7*, *SLC17A6*, *KCNA2*, *KCNK* gene family, *TMEM120A*, *MIDFIC*, *MDFI*, and *MyoD1* genes in ALS-related disease processes. Assessment and categorization of the variants was carried out in compliance with the 2015 joint consensus recommendation of the American College of Medical Genetics and Genomics and genomics using online variant prioritization tools (such as Franklin by genooxa and VarSome—both accessed on 15 November 2024) [90,91].

In addition, we tested the variants detected in our cohort to see whether they can also be found in the project MinE database [48]. The project MinE database was first screened for genes of our interest, and then we analyzed the variants that had a minor allele frequency in ALS patients of 1% or less than that and were absent from control samples. Descriptive statistical analyses were used in our work.

## 5. Limitations

The sample size of the analyzed cohort was fairly low, considering that it stems from a clinic providing care for ALS patients only in a region of a country with a relatively small population. Therefore, the findings of the current study need to be confirmed by future studies on cohorts with a larger sample size, not to mention functional ones.

Another shortcoming of our work is that the above-mentioned genes and their variants do not yet have a fully understood relationship with ALS. Due to the smaller size of our cohort, the reported allele balances may be skewed. Population-specific genetic background of the Hungarian ALS population has been a focus of research in recent years. Our manuscript wishes to contribute to the growing body of information on this topic.

## 6. Conclusions

The results of the current reanalysis may support the theoretical acquired Piezo2 channelopathy-induced ALS onsetting pathogenesis. The found charge-altering variants of *SDC3*, and the variants of *KCNA2*, *KCNK1*, *KCNK16,* and *HSF1* are not only interesting in terms of the downstream progressive die-back initiating pathomechanistic pathway, but might highlight the hierarchical degradation and depletion of ion channels and proteins on route to ALS onset. These findings are also intriguing when we consider the earlier Ca_v_1.3 finding from the same ALS cohort. Namely, the gene therapy of Ca_v_1.3 has gained disease-modifying relevance in another neurological disorder since [92]. Especially, *HSF1* seems to be a promising target from our study, in light of a recent finding that *PIEZO2* has a critical role in the mediation of precise magnetic stimulation [34], as was theorized earlier [8]. Electromagnetic stimulation may arise as a fascinating clinical relevance if we consider that ALS and autoimmune diseases, like celiac disease, rheumatoid arthritis (RA), and systemic lupus erythematosus (SLE), share genetic correlation [93]. The underlying proposed acquired Piezo2 channelopathy and K2P link is especially interesting, not only in ALS, as we highlight in this paper, but has been emphasized in RA [68] and SLE [69] as well. Another meaningful clinical relevance is that the overcoming of the skeletal aspect of the suggested progressively lost ultrafast proton-based proprioceptive signaling may be the reason why soft and exoskeletal robotic-assisted therapeutic exercises could be preventive or positive in ALS [18]. Remarkable that the cable attachment points optimization method is a promising technique for cable-driven exoskeletons [94] to fine-tune this lost ultrafast proprioceptive Piezo2 function. However, it is important to note again that the preliminary nature of the current findings needs to be validated, as future functional studies are at need as well.

## Figures and Tables

**Table 1:** Identified *SDC3* variants of the 21 ALS patients used in the present study.

Gene	Transcript Number	Variant	MAF * in Non-Finnish European Population in Genome Aggregation Database	American College of Medical Genetics and Genomics (ACMG) Classification	Number of Patients
*SDC3*	NM_014654	c.G76C, p.G26R	0	likely benign	1
*SDC3*	NM_014654	c.G286AT, p.A96T	1.4038%	benign	1
*SDC3*	NM_014654	c.G622A, p.V2081	21.9211%	benign	13
*SDC3*	NM_014654	c.G907A, p.D303N	18.8242%	benign	12

* Minor allele frequencies.

**Table 2:** Identified KCNA2 gene and the KCNK gene family variants of the 21 ALS patients used in the present study.

Gene	Transcript Number	Variant	MAF * in non-Finnish European Population in Genome Aggregation Database	American College of Medical Genetics and Genomics (ACMG) Classification	Number of Patients
*KCNA2*	NM_004974.4	c.T1351C, p.S451P	0	VUS leaning pathogenic	1
*KCNK1*	NM_002245.4	c.T2A, p.M1K	0	VUS	1
*KCNK16*	NM_001135106.2	c.C502T, p.Q168 *	0	VUS	1
*KCNK18*	NM_181840.1	c.234del, p.Asp78Glufs*13	0	likely pathogenic	1

* Minor allele frequencies.

**Table 3:** Identified the HSF1 gene variant in the 21 ALS patients used in the present study.

Gene	Transcript Number	Variant	MAF * in Non-Finnish European Population in Genome Aggregation Database	American College of Medical Genetics and Genomics (ACMG) Classification	Number of Patients
*HSF1*	NM_005526.4	c.A861-2C	0	VUS	1

* Minor allele frequencies.

**Table 4:** Pathway analysis.

Pathway	Participating Genes	Mechanistic Role	Potentially ALS-Relevant Consequences
ECM–Receptor Interaction (KEGG hsa04512)	*SDC3*, *VCAN*, *ACAN*	ECM binding, adhesion, mechanosensing	Altered ECM stiffness, disrupted cell–matrix signaling
Focal Adhesion/PI3K–Akt	*SDC3*, *VCAN*, *KCNA2*	Integrin–FAK–Akt–mTOR axis	Cytoskeletal stress, impaired autophagy
Mechanosensation/Piezo–ASIC–KCNK	*SDC3*, *ASICs*, *KCNK* gene family	Integrin–FAK–Akt–mTOR axis	Abnormal excitability, Ca^2+^ influx
Ion Homeostasis/Excitability	*ASICs*, *KCNA2*, *KCNK*	Sensing tension, acid, stretch	Hyperexcitability, excitotoxicity
Neuroinflammation (NF-κB, TNF)	*VCAN* fragments, *ASICs*, *KCNK*	Na^+^/K^+^/Ca^2+^ balance	Microglial activation, motor neuron death
CASK–Syndecan–Kv Channel Complex	*SDC3*, *KCNA2*	ECM degradation → TLR activation	Synaptic instability, altered plasticity

**Table 5:** Baseline characteristics of the 21 Hungarian patients involved in the present study [7].

Minimum age	40
Maximum age	73
Average age	60.0526
Standard deviation	8.8095
Number of females	10
Number of males	11

## Data Availability

The raw sequencing data of the 107 patients analyzed during the current study have been deposited in the NCBI Sequence Read Archive with BioProject accession no. PRJNA549957 (https://www.ncbi.nlm.nih.gov/sra/PRJNA549957—accessed on 15 November 2024).

## Supplementary Materials

The following supporting information can be downloaded at: https://www.mdpi.com/article/10.3390/ijms262010218/s1.

## Abbreviations

The following abbreviations are used in this manuscript:

ALS	amyotrophic lateral sclerosis
CNS	central nervous system
DOMS	delayed-onset muscle soreness
DRG	dorsal root ganglion
ECM	extracellular matrix
GDNF	glial cell line-derived neuropathic factor
GWAS	genome-wide association study
HSF1	heat shock transcription factor-1
HSR1	heat shock RNA-1
MAF	minor allele frequency
MLR	medium latency response
NMDA	N-methyl-D-aspartate
OXPHOS	oxidative phosphorylation
RA	rheumatoid arthritis
REMFS	repeated electromagnetic field stimulation
SLE	systemic lupus erythematosus
SMA	spinal muscular atrophy
SDC3	syndecan-3
VGLUT1	vesicular glutamate transporter 1
VGLUT2	vesicular glutamate transporter 2
VUS	variants of unknown significance
WES	whole exome sequencing
